# HSP70 governs permeability and mechanotransduction in primary human endothelial cells

**DOI:** 10.1002/2211-5463.70129

**Published:** 2025-09-26

**Authors:** Andrea Pinto‐Martinez, Everton G. Melo, Isadora C. B. Pavan, Percíllia V. S. Oliveira, Luiza B. C. T. Coimbra, Thaís L. S. Araujo

**Affiliations:** ^1^ Department of Biochemistry, Institute of Chemistry Universidade de São Paulo Brazil; ^2^ Laboratorio de Biologia Vascular, LIM‐64 (Biologia Cardiovascular Translacional), Instituto do Coração (InCor), Hospital das Clinicas HCFMUSP, Faculdade de Medicina Universidade de São Paulo SP Brazil; ^3^ Department of Organic Chemistry, Institute of Chemistry Universidade Estadual de Campinas ‐ UNICAMP Campinas São Paulo Brazil

**Keywords:** endothelial cell, endothelial cell junctions, HSP70, permeability, shear stress

## Abstract

Vascular barrier disruption is a hallmark of diseases such as cardiovascular disease, stroke, hypertension, pulmonary disorders, infections, and cancer. Endothelium permeability is tightly regulated by shear stress, allowing tissue perfusion, while disturbed flow leads to increased permeability. Cell–cell junctional proteins, including platelet/endothelial cell adhesion molecule‐1 (PECAM‐1)/CD31 and VE‐cadherin, play significant roles in mechanotransduction and barrier integrity. The 70 kDa heat shock protein HSP70 has a well‐established cytoprotective function in cardiovascular physiology. Here, we hypothesized that HSP70 interacts with and regulates these junctional proteins. We found that PECAM‐1 and VE‐cadherin co‐immunoprecipitate with endogenous HSP70, and both proteins exhibited positive proximity ligation assay signals in the endothelial monolayers. HSP70 loss of function leads to disassembly of VE‐cadherin and PECAM‐1 at the cell surface and selectively decreases PECAM‐1 steady‐state expression. Consistent with its vascular protective role, HSP70 inhibition also reduced endothelial nitric oxide synthase (eNOS) levels. Furthermore, HSP70 was essential for maintaining normal paracellular flux in primary vein (HUVEC) and coronary artery endothelial cells (HCAEC) monolayers, as well as for promoting natural cell alignment under physiological laminar shear stress in HUVEC. These results demonstrate that HSP70 regulates the quality control of interendothelial adherens junctions, mediates responses to hemodynamic forces, and maintains monolayer barrier function across vascular beds. Our findings advance the mechanistic understanding of how human HSP70 mediates vascular homeostasis through endothelium responses to blood flow and permeability in addition to HSP70 role in migration, proliferation, and angiogenesis.

## Graphical abstract

HSP70 chemical inhibition reduces endothelial cell proliferation and increases permeability, the latter supported by normal interendothelial junctional protein distribution. HSP70 also plays a role in shear stress response, a hemodynamic force naturally present in blood vessels and correlated with vessel protection. Our work presents a new perspective centered on HSP70 as a major contributor to the healthy human endothelial monolayer.

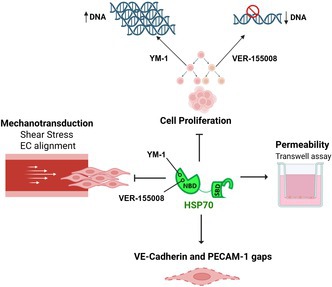

**Abbreviations**
CTEPH: chronic thromboembolic pulmonary hypertension
EC: endothelial cells
eNOS: endothelial nitric oxide synthase
HCAEC: human coronary artery endothelial cells
HSP70: 70 kDa heat shock protein
HUVEC: human vein endothelial cells
PECAM‐1: platelet/endothelial cell adhesion molecule‐1
TM: thrombomodulin
VEGF: vascular endothelial growth factor
VEGFR‐2: vascular endothelial growth factor receptor

Endothelial dysfunction due to proteostasis imbalance is a hallmark of cardiovascular diseases such as thrombosis, atherosclerosis, atrial fibrillation, and myocardial ischemia–reperfusion injury [1]. One well‐characterized form of endothelium dysregulation is the loss of monolayer continuity through disruption of interendothelial contacts mediated by adherens junction proteins and tight junctions [2], a phenomenon known as vascular hyperpermeability [3]. In addition to cardiovascular diseases, impaired permeability control is commonly observed in stroke, cancer, diabetes, infections, pulmonary conditions such as asthma, and hematologic diseases like sickle cell disease [4].

The blood flow generates a hemodynamic force known as shear stress, which determines the endothelial cell phenotype characterized by either a protective laminar flow or a proatherogenic oscillatory/turbulent flow [5]. The latter prevents the expected cell alignment in the direction of flow. Mechanotransduction is initiated by several mechanosensors including ion channels, G protein‐coupled receptors, and adhesion proteins such as integrins [6]. One crucial multi‐protein mechanosensory complex consists of platelet/endothelial cell adhesion molecule‐1 (PECAM‐1)/CD31, VE‐cadherin, and vascular endothelial growth factor receptor (VEGFR‐2) [7]. PECAM‐1, a 130 kDa glycoprotein, is an essential endothelial mechanosensory protein that allows transendothelial leucocyte migration through a homophilic interaction that controls inflammation [8]. Furthermore, PECAM‐1 contributes to endothelial migration, survival, and angiogenesis, and as localized deep in the endothelial cell junctional, it plays a positive role in vascular permeability. VE‐cadherin is an adherens junctional protein that regulates vascular permeability through adhesion properties [2, 8]. Mouse endothelial cells lacking VE‐cadherin lose the capacity for flow‐induced cell alignment, which is restored by full VE‐cadherin expression, demonstrating the central importance of this junctional protein in mechanotransduction. VE‐cadherin serves as an adaptor by directly binding to VEGFR‐2 [9], consistent with findings that its turnover directly affects its function [10].

The 70‐kDa heat shock protein family, HSP70, exerts a central function in maintaining proteostasis by supporting productive multidomain protein folding and assembly [11]. HSP70 is upregulated by environmental stresses such as heat [12], shear stress [13, 14], oxidative stress, low oxygen pressure (hypoxia) and infection [15]. HSP70 and HSP90 are downregulated in endothelial cells (EC) of patients with chronic thromboembolic pulmonary hypertension (CTEPH), in line with the abrogation of pulmonary physiological shear stress upregulation [14]. Low levels of HSP70 are found in human EC monolayers and mouse aortas exposed to proatherogenic flow [13]. Loss of HSP70 function impairs EC migration [14, 16, 17], IL‐5‐stimulated endothelial nitric oxide synthase (eNOS) phosphorylation [18], nucleolin surface translocation [17], basal expression of phosphatidylinositol 3‐kinase (PI3K) p110 subunit [19], and vascular endothelial growth factor (VEGF)‐ or asymmetric dimethylarginine‐mediated‐AKT phosphorylation [19, 20]. The chaperome, formed by HSP70, other heat shock proteins, cochaperones, and cofactors, constitutes a significant portion of the total protein mass in human cells [21] and binds large polypeptide chains.

We discovered two new HSP70 likely clients: PECAM‐1/CD31 and VE‐cadherin. Additionally, other vascular proteins such as thrombomodulin (TM) and eNOS point to HSP70 as a major regulator of EC function. Cell‐surface thrombomodulin remains unchanged with HSP70 inhibition, except that ER‐to‐Golgi trafficking is blocked by VER‐155008, consistent with a similar pattern observed under heat shock conditions. These results open new avenues to treat several vascular diseases characterized by hyperpermeability, such as asthma, ischemic stroke, and infection. Enhancing chaperone function is a promising therapeutic strategy to be investigated, either by increasing levels of HSP70 or by activating its chaperone activity. This approach may help restore PECAM‐1 and VE‐cadherin expression, closing intercellular gaps and ultimately recover vessel integrity in disease states. Moreover, loss of mechanotransduction could be related to atherosclerosis biogenesis, and increasing HSP70 expression specifically in dysfunctional endothelial cells could represent a strategy to treat or slow the disease progression.

## Materials and methods

### Cell culture

Primary human endothelial cells obtained from Lonza (were cultured in a humidified incubator at 37 °C and 5% CO_2_ (Thermo Scientific and CELLXPERT^®^C170I, Eppendorf) in adherent culture flasks with EBM‐2 (CC‐3156; Lonza, Walkersville, MD, USA) supplemented with the EGM‐2 BulletKit (CC‐3162; Lonza) for human umbilical vein endothelial cells (HUVECs, passages 2–5, C2519A, Lonza) or the EGM‐2MV BulletKit for human coronary artery endothelial cells (HCAECs, passages 4–7, CC‐2585; Lonza). Confluent adherent cells were washed with 25 mM HEPES buffer (pH 7.5), detached with 0.25% trypsin/EDTA, and subcultured. All experiments were performed in EBM‐2 medium supplemented with EGM‐2 or EGM‐2MV, unless otherwise specified, using the different cell lots described in Table 1. VER‐155008 and YM‐1 concentration used was based on cytotoxicity and proliferation assays previously published [12].

**Table 1:** HUVEC and HCAEC lots.

Cell	Brand	Lot number	Assays
HUVEC	Lonza	23TL213682 23TL135742	Permeability
HUVEC	Lonza	21TL95720 23TL104314	Western blotting
HUVEC	Lonza	23TL213682 23TL086130	Immunoprecipitation
HUVEC	Lonza	19TL028323 21TL95720	Click Edu Alexa
HUVEC	Lonza	19TL028323 23TL135742	PLA
HUVEC	Lonza	19TL028324 19TL023264 23TL213682 23TL086130 21TL195720	IF
HCAEC	Lonza	21TL316169 23TL213682	Shear Stress Permeability IF

### Click it Edu DNA synthesis assays

To evaluate cell cycle progression and detect S phase entry, primary human umbilical vein endothelial cells (HUVECs) obtained from Lonza were cultured in 6‐well plates at a density of 2 × 10^5^ cells per well. Cells were maintained in EGM‐2 endothelial growth medium (Lonza) and allowed to adhere and proliferate for 24 h. Subsequently, cells were treated with either vehicle control (0.1% DMSO), VER‐155008 (30 μm), or YM‐1 (20 μm) for an additional 24 h. Unstained cells served as a negative control. After treatment, cells were trypsinized, collected, and processed using the Click‐iT™ EdU Alexa Fluor™ 488 Flow Cytometry Assay Kit (Thermo Fisher Scientific), following the manufacturer's protocol. Briefly, cells were incubated with 10 μm EdU for 1 h at 37 °C to label actively replicating DNA. Following EdU incorporation, cells were fixed, permeabilized, and stained via a copper‐catalyzed click reaction to conjugate Alexa Fluor™ 488 to the incorporated EdU. Samples were analyzed using a BD Accuri™ C6 Plus flow cytometer equipped with a CSampler™ autosampler. A total of 50,000 events were acquired per sample at a flow rate of approximately 50,000 events per second. Gating for EdU‐positive (S phase) cells was defined using the unstained and vehicle‐treated controls.

### Shear stress

Primary HUVEC were plated in 100 mm culture dishes plates at 3.5 × 10^6^ cells in EBM‐2 supplemented with EGM‐2 for 6 h in a humidified incubator at 37 °C and 5% CO_2_. Next, cells HEPES‐washed were 18h starved in EBM‐2 with 0.2% serum fetal bovine (SFB, Gibco) treated or not with 50 μm VER‐155008. Then, cells in EBM‐2 supplemented with EGM‐2 were submitted to 15 dynes per cm^2^ for 24h treated or not with 50 μm VER‐155008 in a cone‐and‐plate system, when indicated static conditions cells were not submitted to shear stress. Optical microscopy images of three independent experiments. HCAECs were submitted to equal shear stress after 24 h of growth. Primary HCAEC were plated in 100 mm culture dish plates at 2.7 × 10^6^ cells in EBM‐2 supplemented with EGM‐2 MV for 12 h in a humidified incubator at 37 °C and 5% CO_2_. Next, cells HEPES‐washed were 4 h starved in EBM‐2 treated or not with 30 μm VER‐155008 in the last hour. Then, the cell medium was replaced with EGM‐2‐MV, and cells were exposed to 15 dynes per cm^2^ (corresponding to 438 RPM) for 24 h with or without 30 μm VER‐155008 using a cone‐and‐plate system [5, 13, 14, 22]. Five predefined regions of the culture dish were imaged in an optical microscope before and after the shear exposure in each condition. A representative image of five independent experiments is shown.

### Endogenous HSC70 immunoprecipitation

Primary HUVEC were plated in 100 mm plates at 0.3 × 10^6^ cell for 7 days in basal medium EBM‐2 supplemented with EGM‐2, with medium changed every 2 days, HEPES buffer washed, and scraper detachment followed by centrifugation (1500 ***rpm***, 10 °C, for 5 min). The pellet was resuspended in 150 μL lysis buffer (50 mm Tris‐base pH 7.5, 150 mm NaCl, 1 mm EDTA, 1% Triton, and 1 : 100 protease and 1 : 100 phosphatase inhibitors). The lysate was under agitation on ice for 20 min after being centrifuged (12,000 ***rpm***, 4 °C, 10 min), and the supernatant was collected. According to the manufacturer's instructions, protein concentration was quantified using the bicinchoninic acid (BCA) assay. About 700 μg of protein were incubated with rat IgG control and endogenous HSC70 immunoprecipitation as follows. Lysates were incubated overnight at 4 °C with 1.3 μg of rat IgG isotype antibody or HSC70 antibody. Lysate was incubated with 50 μL of the A/G agarose beads (Tris base pH 7.5 and 150 mm NaCl) for 3 h at 4 °C with agitation. Previously, 50 μL of A/G agarose beads were washed five times with 25 mm Tris base pH 7.5 and 150 mM NaCl by centrifugation at 8200 × ***g*** for 1 min and on ice for 2 min. After, samples were washed five times with 500 μL wash buffer (Tris base pH 7.5 and 150 mm NaCl), and protein co‐immunoprecipitated was eluted with 50 μL of sample buffer (12.5 mm Tris pH 6.8, 1.25% beta‐mercaptoethanol, 0.5% SDS, 0.0125% bromophenol blue, and 2.5% glycerol) and boiled at 100 °C for 10 min. Finally, 15 μL of the immunoprecipitation eluted and 30 μg of total protein extract (input) were resolved by SDS/PAGE, transferred onto nitrocellulose membranes, and probed with primary antibodies listed in Table 2 diluted in TBST 0.1% HSC70, VE‐cadherin, and PECAM‐1 at western blotting. Next, membranes were incubated with infrared fluorescent secondary antibodies: IRDye^®^ 800CW Donkey anti‐Mouse IgG and IRDye^®^ 680RD Donkey anti‐Rabbit IgG. Blots were scanned using LI‐COR OdysseyR 2‐channel near‐infrared fluorescent imager imaging System (Odyssey DL‐x) and visualized protein bands quantified according to pixel density with Empiria Studio as a manufacturer's recommendation.

**Table 2:** Antibodies used in experimental assays.

Target/antibody	Brand	Catalog number	Assay
HSP70	Invitrogen	MA3‐006	WB 1:1000, IF 1:400, PLA
Inducible HSP70	Invitrogen	MA3‐009	WB 1:1000
HSP90	Cell signaling	4877	WB 1:1000
HSP40	Cell signaling	4868	WB 1:500
HSC70	Invitrogen	PA5‐27337	WB 1:1000, PLA
HSC70	Invitrogen	MA1‐26078	IP 1.3 μg
VE‐cadherin	Cell signaling	2500	WB 1:1000, IF 1:400, IP, PLA
VE‐cadherin	Abcam	ab33168	IF 1:400
PECAM‐1	Cell signaling	3528	WB 1:1000, IF 1:200, IP, PLA
Thrombomodulin	Invitrogen	PA5‐21924	IF 1:200
GM130	BD Biosciences	610 822	IF 1:600
eNOS	Cell signaling	32027	WB 1:1000
β‐Actin	Sigma‐Aldrich	A5441	WB 1:10.000
Goat anti‐Rabbit IgG (H + L) Secondary Antibody	Invitrogen	31 460	Permeability
Rat IgG – Isotype control	Abcam	ab18450	IP 1.3 μg
Anti‐Mouse IgG Secondary Antibody	LI‐COR	926–32 212	WB 1:10.000
Anti‐Rabbit IgG Secondary Antibody	LI‐COR	926–68 073	WB 1:10.000
Alexa Fluor 488	Invitrogen	2 110 499	IF 1:400
Alexa Fluor 546	Invitrogen	A11030	IF 1:400

IF, Immunofluorescence; IP, Immunoprecipitation; WB, western blot.

### Western blotting

About 20–40 μg of total protein lysates were subjected to SDS/PAGE using a 12% bis‐acrylamide gel at 100 V at room temperature in running buffer (25 mm Tris‐Base, 192 mm glycine, and 1% sodium dodecyl sulfate (SDS), pH 8.3) using a Bio‐Rad system (mini‐PROTEAN Tetra Cell and PowerPac Basic Power Supply). The obtained gel was then transferred to a membrane using wet transfer (Mini Trans‐Blot Module Core, Bio‐Rad) at 100 V at 4 °C, with transfer buffer containing 48 mm Tris‐Base, 39 mm glycine, 0.037% SDS, and 20% methanol, pH 8.3. The nitrocellulose membrane was blocked with 5% nonfat milk in TBS‐T (20 mm Tris‐Base, 150 mm NaCl, and 0.1% Tween‐20, pH 7.4) for 1 h at room temperature and then incubated with primary antibody solution in TBS‐T overnight at 4 °C. The following day, the membrane was washed three times with TBS‐T for 10 min and incubated with secondary antibodies: IRDye^®^ 800CW Donkey anti‐Mouse IgG or (most)/and IRDye^®^ 680RD Donkey anti‐Rabbit IgG diluted 1 : 10000 in TBS‐T, for 1.5 h at room temperature. This protocol is based on previous work [13]. The membrane was washed three times with TBS‐T for 10 min before membranes were scanned using LI‐COR OdysseyR 2‐channel near‐infrared fluorescent imager imaging System (Odyssey DL‐x) and visualized protein bands quantified according to pixel density with Empiria Studio or ImageJ as manufactured recommendation. The specifications of the antibodies used are listed in Table 2 and full material and reagents in Table 3.

**Table 3:** Materials and reagents.

Material or reagent	Brand	Catalog number
Pierce™ BCA Protein Assay Kits	Thermo Fisher Scientific	23 227
Acrylamide/Bis‐acrylamide, 30%	Sigma‐Aldrich	A3574
Click‐iT (Plus EdU Alexa Fluor 488)	Invitrogen	C10633
Lonza Endothelial Basal Medium‐2 (EBM‐2)	Lonza	CC‐3156
Bullet kit EGM‐2	Lonza	CC‐3162
Bullet kit EGM‐2 MV	Lonza	CC‐3202
Halt™ Protease Inhibitor Cocktail (100X)	Thermo Fisher Scientific	78 429
Halt™ Phosphatase Inhibitor Cocktail (100X)	Thermo Fisher Scientific	78 420
75cm^2^ U‐Shaped Canted Neck Cell Culture Flask	Corning	3290
100 mm Culture Dish	Corning	430 167
Fetal Bovine Serum, Qualified, Brazil (SFB)	Gibco	12 657 029
Pierce™ Protein A/G Agarose	Thermo Scientific	20 421
Millicell^®^ Cell Culture Insert	Sigma‐Aldrich	PIHP01250
Corning^®^ ELISA Microplates	Corning	3369
ELISA Buffer Kit	Invitrogen	CNB0011
Glycerol	Sigma‐Aldrich	G5516
HEPES	Sigma‐Aldrich	H3375
Nitrocellulose Membrane	Bio‐Rad	1 620 115
Methanol	Merck	106 007
Trizma Base	Sigma‐Aldrich	93 352
VER‐155008	Sigma‐Aldrich	SML0271
YM‐1	Sigma‐Aldrich	SML0943
EZ Slide Millicell	Sigma‐Aldrich	PEZGS0816
NP‐40	Sigma‐Aldrich	I8896
Bovine Serum Albumin (BSA)	Sigma‐Aldrich	A7906
Hoechst	Sigma‐Aldrich	B2261
ProLong™ Diamond Antifade Mountant	Invitrogen	P36961
Triton™ X‐100	Sigma‐Aldrich	T8787
Sodium chloride (NaCl)	Sigma‐Aldrich	59 888
Dibasic sodium phosphate (Na_2_HPO_4_)	Synth	F103201
Monobasic potassium phosphate (KH_2_PO_4_)	Sigma‐Aldrich	P9791
Potassium chloride (KCl)	Sigma‐Aldrich	104 936
Glycine	Sigma‐Aldrich	G8898
Sodium dodecyl sulfate (SDS)	Sigma‐Aldrich	L3771
Tween‐20	Sigma‐Aldrich	P1379
DMSO	Sigma‐Aldrich	D8418

### Permeability assay

The permeability assay was described previously [23]. HUVEC seeded at 2.5 × 10^5^ cells in 400 μL EGM‐2 per Millicell Transwell inserts in a 24‐well plate bottom with 500 μL of EGM‐2, followed by 48 h at 37 °C, 5% CO_2_. The medium was changed by EBM‐2 and treated with 50 μm VER‐155008 and 40 μm YM‐1 for 1 h at 5% CO_2_ and 37 °C. The two controls were performed: (1) a control without cells, corresponding to the total antibody passage control, and (2) a control with cells treated with DMSO, VER‐155008 diluent. 16 ng·mL^−1^ Goat anti‐Rabbit IgG (H + L) Secondary Antibody‐HRP upper insert. A volume of 10 μL of the medium outside the insert was collected at 0, 15, and 30 min in quadruplicate and transferred to an ELISA plate, followed by 100 μL of TMB for 30 min at 25 °C. Next, 100 μL of stop solution was added to each well, and absorbance (Synergy H1 hybrid reader‐Biotek) was measured at 450 nm. The experiments were conducted three times independently using cells from different passages. Data were analyzed using statistical analyses described below.

### Immunofluorescence

Human coronary artery endothelial cells (HCAECs) or human vein endothelial cells (HUVECs) were cultured on Millicell EZ Slides (Merck Millipore, Darmstadt, Germany) and treated under three different conditions: basal DMSO (vehicle control), 30 μm VER‐155008, and 20 μm YM‐1. After 24 h of treatment, cells were fixed with 4% paraformaldehyde for 20 min at room temperature and permeabilized with 0.1% NP‐40 in PBS for 30 min at 37 °C. Nonspecific binding was blocked using 2% bovine serum albumin (BSA) in PBS for 30 h at 37 °C [12]. Cells were incubated overnight at 4 °C with the following primary antibodies diluted in 1% BSA buffer: rabbit anti‐VE‐cadherin (1 : 400; Abcam, ab33168) or rabbit anti‐VE‐cadherin (1 : 400; Cell Signaling Technology, 2500) and mouse anti‐PECAM‐1 (1 : 200; Cell Signaling Technology, 3528S). After several washes, cells were incubated for 1 h at room temperature with the appropriate secondary antibodies: Alexa Fluor 488‐conjugated goat anti‐rabbit IgG and Alexa Fluor 546‐conjugated goat anti‐mouse IgG (Thermo Fisher Scientific, Waltham, MA, USA), both diluted 1 : 400. Nuclei were counterstained with Hoechst. Coverslips were mounted using ProLong™ Gold Antifade Mountant (Thermo Fisher Scientific), and samples were imaged using a Leica TCS SP8 confocal microscope (Leica Microsystems, Wetzlar, Germany) in the INFAR‐UNIFESP facility. Image acquisition and analysis were performed using LAS X software (Leica Microsystems).

### Proximity ligation assay (PLA)

Protein–protein interactions were evaluated using the Duolink^®^ *In Situ* Red Starter Kit (Sigma‐Aldrich, St. Louis, MO, USA), following the manufacturer's protocol. Human Umbilical Vein Endothelial Cells (HUVEC) were seeded onto the wells of a Millicell EZ slide (Merck Millipore, Darmstadt, Germany) at a concentration of 4 × 10^4^ cells per well and cultured for 24 h. Cells were fixed with 4% paraformaldehyde for 20 min and permeabilized with 0,1% NP‐40 for 30 min at 37 °C. After removing the detergent, nonspecific binding was blocked using BSA 2% in PBS for 30 min at 37 °C. To evaluate specific protein interactions, cells were incubated overnight at 4 °C with primary antibody pairs, targeting either HSC70 and PECAM‐1 (rabbit anti‐HSC70 PAS‐27337 and mouse anti‐PECAM‐1 Cell Signaling 35 288) or HSP70 and VE‐cadherin (rabbit anti‐VE‐cadherin abcam EPR 27436–55 and mouse anti‐HSP70 abcam ab47455). After washing with PBS, PLA probes (anti‐rabbit PLUS and anti‐mouse MINUS) were added and incubated for 1 h at 37 °C. After washing, the ligation and amplification steps were performed as indicated in the kit's protocol. Nuclei were stained with DAPI, and coverslips were mounted using Prolong™ Gold Antifade Mountant (Thermo Fisher Scientific, Waltham, MA, USA). Samples were imaged using a confocal microscope (Leica DMi8) under identical acquisition settings in the INFAR‐UNIFESP facility. For each condition, at least five random fields per well were analyzed in three independent experiments.

### Statistics

Graphs were generated and statistical analyses were performed using GraphPad Prism software. For comparisons involving multiple groups, one‐way ANOVA followed by Tukey's *post hoc* test was used, and for comparisons of two groups, the t test was used. A *P*‐value of less than 0.05 was considered statistically significant, with significance described as *P < 0.05, **P < 0.01, and ***P < 0.001. All the results are shown as error bars indicating the mean ± SEM and were validated through a minimum of three independent experiments.

## Results

**Fig. 1:**
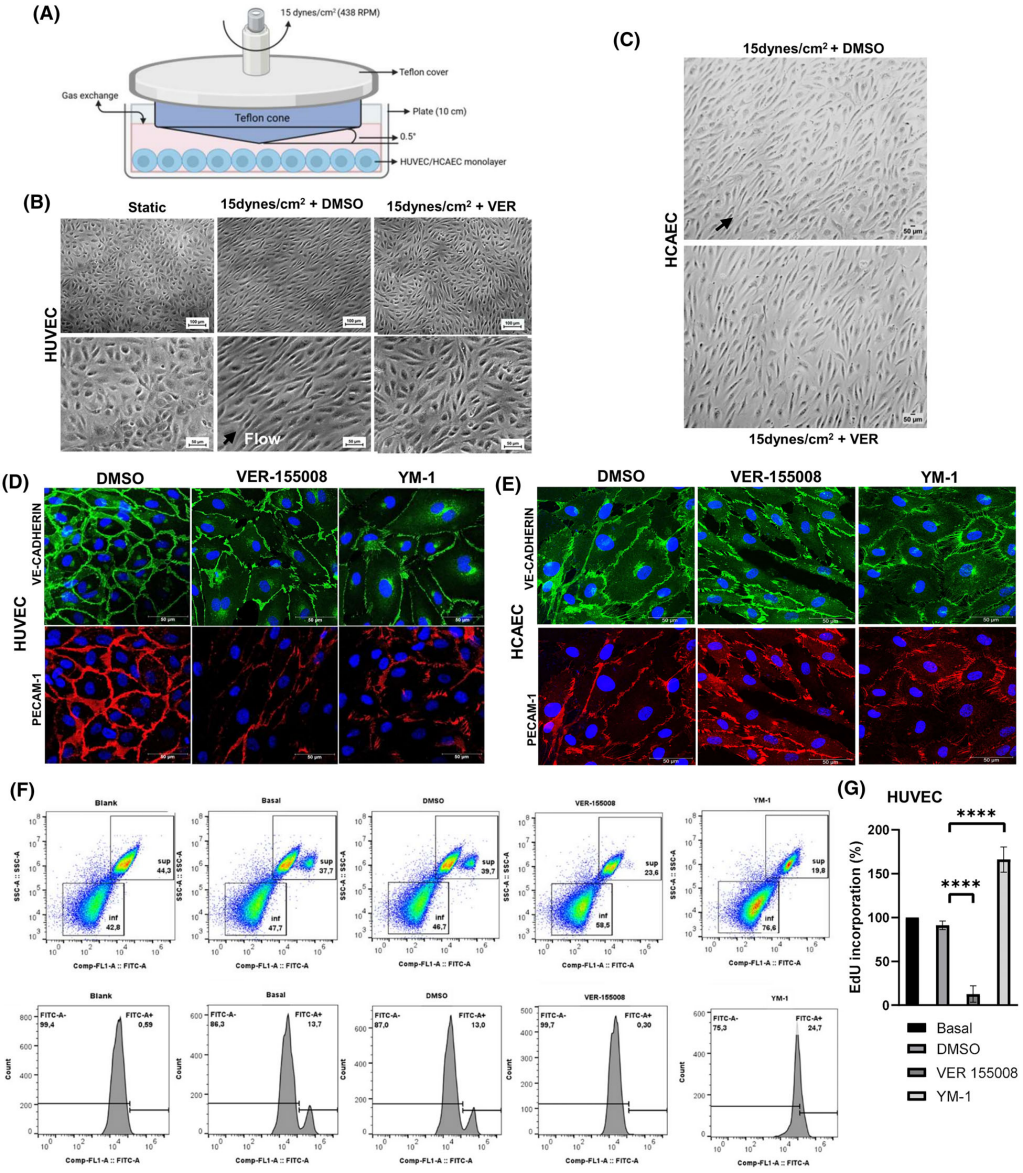
HSP70 alters the expression pattern of intercellular junctional proteins and endothelial phenotype in primary human endothelial cells. (A) Schematic representation of the shear stress cone‐and‐plate system. In this setup, HUVEC or HCAEC were exposed to laminar flow due to the rotation of a Teflon cone, with a fixed angle of 0.5° from the center to the edge, over the cell culture medium in a humidified incubator at 37 °C and 5% CO_2_. (B) Primary HUVEC pretreated with 50 μm VER‐155008 for 1 h, followed by exposure to 15 dynes per cm^2^ laminar shear stress for 24 h in the presence or absence of 50 μm VER‐155008 or under static conditions. Images are representative of three independent experiments. Scale bar superior: 100 μm and inferior panel: 50 μm (C) Primary HCAEC pretreated with 30 μm VER‐155008 for 1 h followed by exposure to 15dynes per cm^2^ laminar shear stress for 24 h in the presence or absence of 30 μm VER‐155008. Images are representative of five independent experiments. Scale bar: 50 μm. (D) Confocal immunofluorescence images of VE‐Cadherin (green; CST 2500), PECAM‐1 (red, CST 3528), nuclei (blue, Hoechst) of HUVEC and (E) Confocal immunofluorescence images of VE‐Cadherin (green; CST 2500), PECAM‐1 (red, CST 3528), nuclei (blue, Hoechst) of HCAEC in basal conditions or subjected to 24‐h treatment with 30 μm VER‐155008 or 20 μm YM‐1. Scale bars: 50 μm. Images represent three independent experiments from cell lots (21TL316169 and 23TL213682), specified in Table 1. (F) Representative flow cytometry histograms (FITC channel) show EdU‐negative (−) and EdU‐positive (+) populations in HUVEC from three independent experiments: nonlabeled (unstained control), EdU‐stained basal (untreated), DMSO (vehicle control), VER‐155008 (30 μm), and YM‐1 (20 μm). (G) Quantification of EdU incorporation is shown in the bar graph, representing the mean ± SD of normalized data from three independent experiments, from different cell lots (21TL95720, 21TL95720, and 19TL028323). ****P < 0.0001 by one‐way ANOVA followed by post hoc Tukey test.

HSP70 supports endothelial cell functions such as migration and angiogenesis in human umbilical vein endothelial cells (HUVEC) [12, 19] and primary human pulmonary artery endothelial cells (HPAEC) [14]. Further, several heat shock proteins are downregulated in patients, and cytosolic HSP70 is directly associated with endothelial dysfunction observed in type IV hypertension [14]. We hypothesized that HSP70 plays an essential role in the response to hemodynamic forces due to its upregulation expression by physiological shear stress in vein endothelial cells [13] and pulmonary artery endothelial cells [14]. We used an established cone‐and‐plate system (Fig. 1A) to expose HUVEC or HCAEC to laminar flow generated by the rotation of a Teflon cone with a fixed angle of 0.5° angle from the center to the edge, over the cell culture medium, in a humidified incubator at 37 °C and 5% CO_2_ [5, 6]. Interestingly, we showed that chemical HSP70 inhibition precludes the natural alignment of endothelial cells in the direction of physiological flow in primary HUVEC (Fig. 1B) and not affects cell alignment in coronary artery endothelial cells (HCAEC) (Fig. 1C). Both vein and artery endothelial cells were elongated as response to shear stress (Fig. 1B,C), as expected [6]. VER‐155008, a well‐established competitive ATP inhibitor [24, 25, 26], and YM‐1, an allosteric inhibitor [27, 28], were used to chemically inhibit specific HSP70 family members.

Next, we evaluated whether HSP70 affects junctional endothelial proteins, which compose mechanosensory complexes. We discovered that HSP70 loss of function triggers the disassembly of PECAM‐1/CD31 and VE‐cadherin from the cell surface in HUVEC (Fig. 1D) and HCAEC (Fig. 1E). In cancer cells, HSP70 affects the cell cycle and causes aberrant mitosis via high molecular weight complexes named context‐dependent epichaperomes [29]. We evaluated mitosis in vascular cells based on the typical anti‐proliferative effects [12] and across other cell types HSP70 pharmacological inhibition [26]. We showed that VER‐155008 decreases thymidine incorporation, while YM‐1 increases it (Fig. 1F,G). In both Basal and DMSO conditions, the EdU‐positive subpopulation is consistently present, reflecting the baseline proliferative activity of HUVEC in culture. In contrast, VER‐155008 treatment resulted in a fluorescence distribution nearly identical to the blank, suggesting marked suppression of DNA synthesis. YM‐1 treatment, meanwhile, produced a single, broad right‐shifted peak, likely reflecting a population‐wide increase in EdU incorporation or S phase accumulation, indicative of altered DNA replication dynamics.

**Fig. 2:**
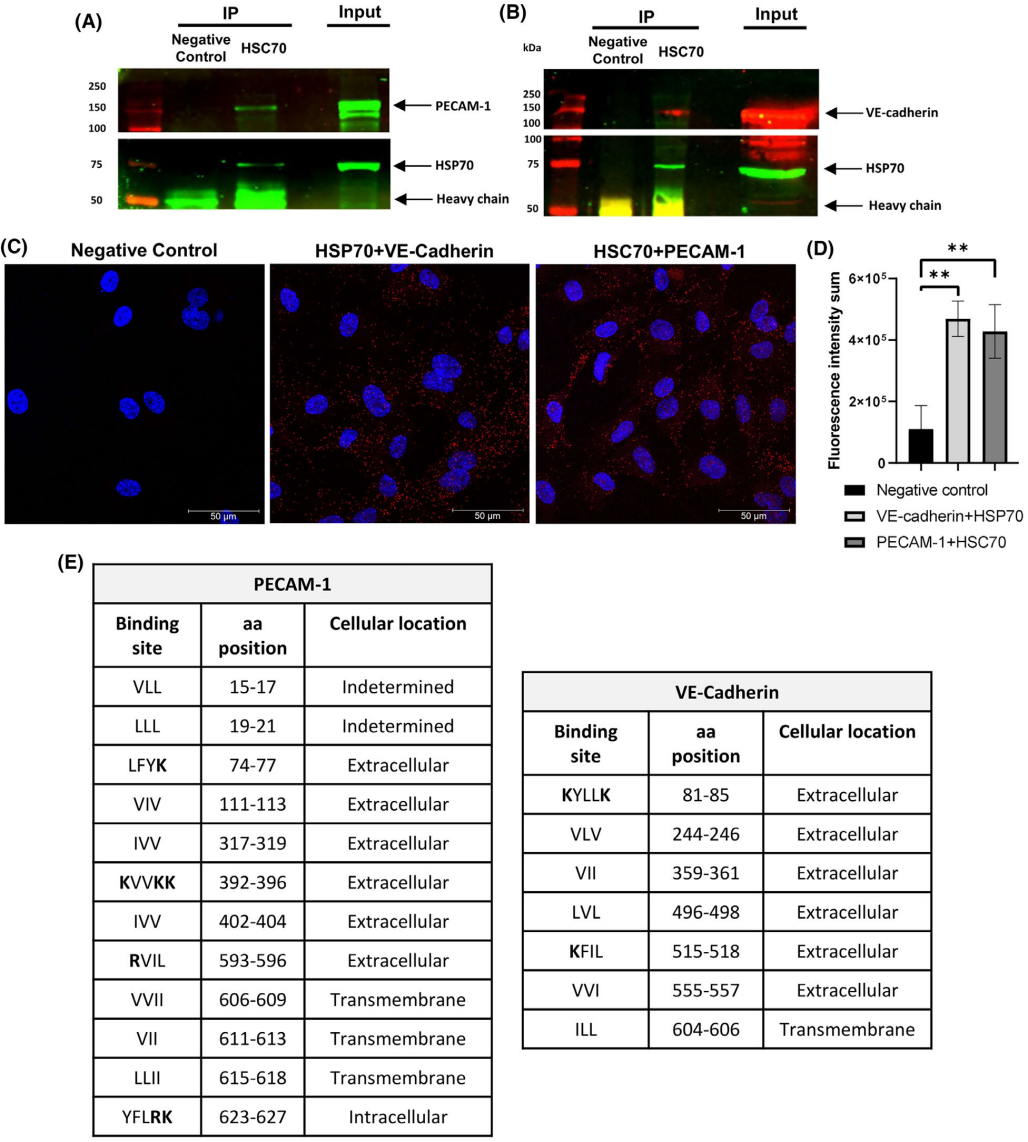
HSP70 interacts with human PECAM‐1 and VE‐cadherin in vascular cells. The total lysate of primary HUVEC cells was collected and subjected to immunoprecipitation with anti‐HSC70 antibody (Invitrogen, MA1‐26078). Rat anti‐IgG antibody (Abcam, ab18450) was used as a control. Membrane probed against (A) HSC70 and PECAM‐1 or (B) VE‐cadherin and HSC70 were assessed through western blotting. Images are representative of three independent experiments using two different lots of cells (23TL213682 and 23TL086130). (C) Confocal image of cells labeled with PLA probes without primary antibodies (negative control), showing interaction between HSP70 (Invitrogen, MA3‐006) and VE‐cadherin (Cell signaling, 2500) and between HSC70 (Invitrogen, PA5‐27337) and PECAM‐1 (Cell signaling, 3528). Cells were incubated with the indicated primary antibodies (except in the negative control), followed by secondary antibodies conjugated with oligonucleotides for proximity ligation assay (PLA). Nuclei were stained with Hoechst (blue). Scale bars, 50 μm. Images are representative of three independent experiments. (D) The sum of fluorescence intensity within each region of interest (ROI) of identical area was measured to quantify the PLA signal. ROIs (red oval outlines) defined in each image from three independent experiments (*n* = 3) performed using different HUVEC lots (23TL135742, 19TL028323, and 23TL135742). Results are presented as mean ± SEM and were analyzed using one‐way ANOVA followed by Tukey's *post hoc* test. Both PLA conditions showed significantly higher signals compared to the negative control (*P* < 0.05). (E) Potential binding sites of HSP70 in VE‐cadherin and PECAM‐1.

**Fig. 3:**
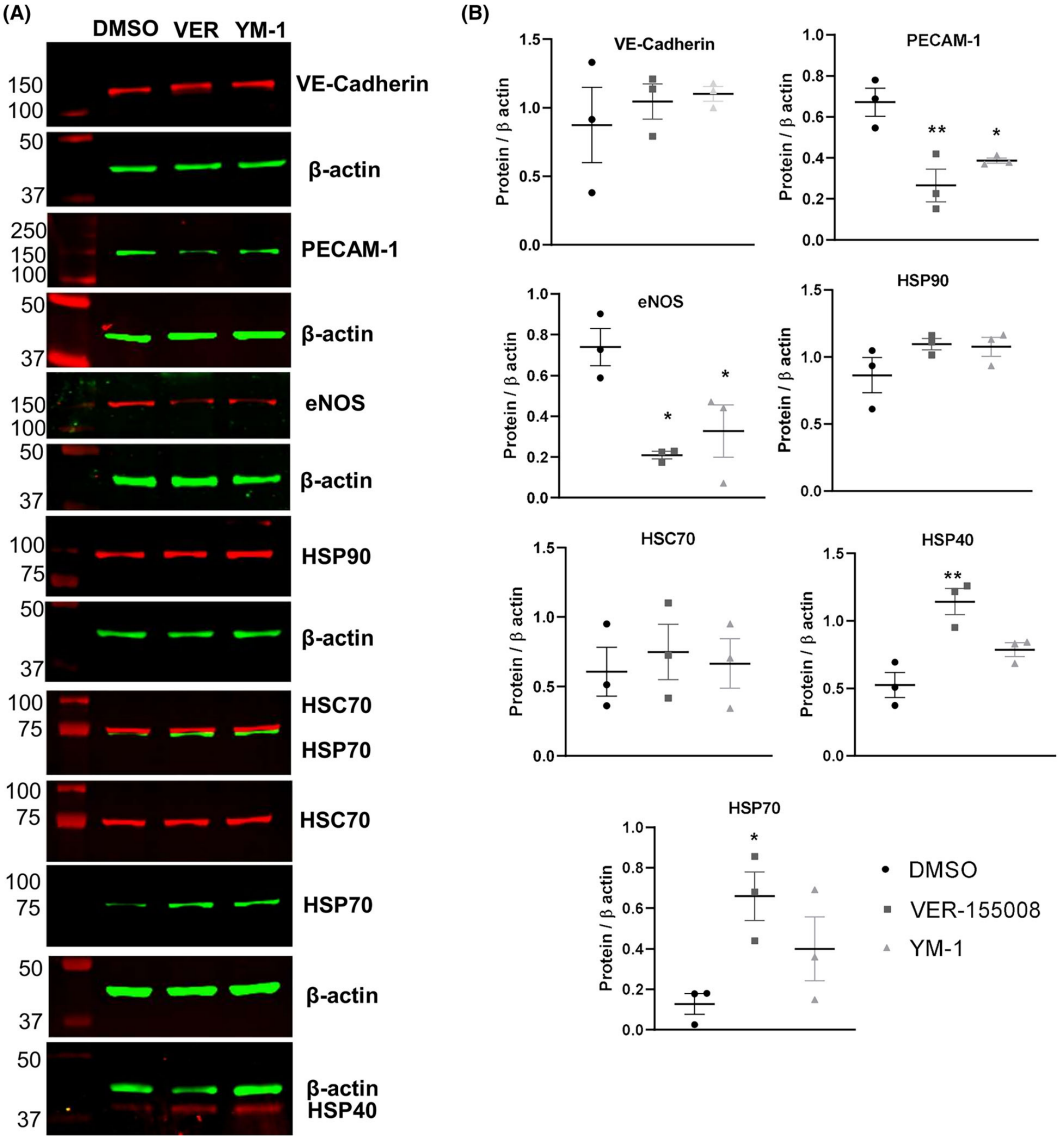
HSP70 controls expression of PECAM‐1 and eNOS in primary human endothelial cells. (A) HUVEC were treated with 30 μm VER‐155008 and 20 μm YM‐1 for 24 h and whole cell lysates were subjected to western blot analysis for VE‐cadherin (CST, 2500), PECAM‐1 (CST, 3528), HSP90 (CST, 4877), HSP70 (Invitrogen, MA3‐009), HSC70 (Invitrogen, PA5‐27337) and eNOS (CST, 32027) of three independent experiments. (B) Data are shown as mean ± SEM of three independent experiments using two different lots of cells (21TL95720 and 23TL104314), one‐way ANOVA, with Tukey's post hoc *n* = 3, *P < 0.05, **P < 0.01 versus Basal.

Based on previous results, we ask whether endothelial junctional proteins are HSP70 interactors. Interestingly, immunoprecipitation of endogenous HSP70 pulls down VE‐cadherin and PECAM‐1 in human ECs (Fig. 2A,B), and proximity ligation assay (PLA) confirmed interaction (Fig. 2C,D). We searched for potential HSP70‐binding sites in PECAM‐1 and VE‐cadherin based on hydrophobic sequences recognized by prototypical HSP70 from *E.coli*, DnaK, in substrates [30]. Notably, the entire protein sequence was analyzed due to the well‐known interaction of HSP70 with nascent polypeptide chains [31]. We observed several potential sites in both PECAM‐1 and VE‐cadherin (Fig. 2E), based on DnaK substrate recognition [31]. The intracellular regions are likely targets considering interaction with the native folded state of the protein, possibly preserved in the previous immunoprecipitation assays. The exact HSP70‐binding sites within the junctional and adhesive proteins will be studied in the future. To further explore the mechanistic function of HSP70 in these new interactors, we evaluated the steady‐state expression of these adhesion proteins. VER‐155008 and YM‐1 reduced PECAM‐1 levels, while VE‐cadherin was barely affected (Fig. 3A,B). As expected under proteotoxic stress, HSP70 and HSP40 were upregulated following HSP70 chaperone inhibition for 24 h (Fig. 3A,B).

**Fig. 4:**
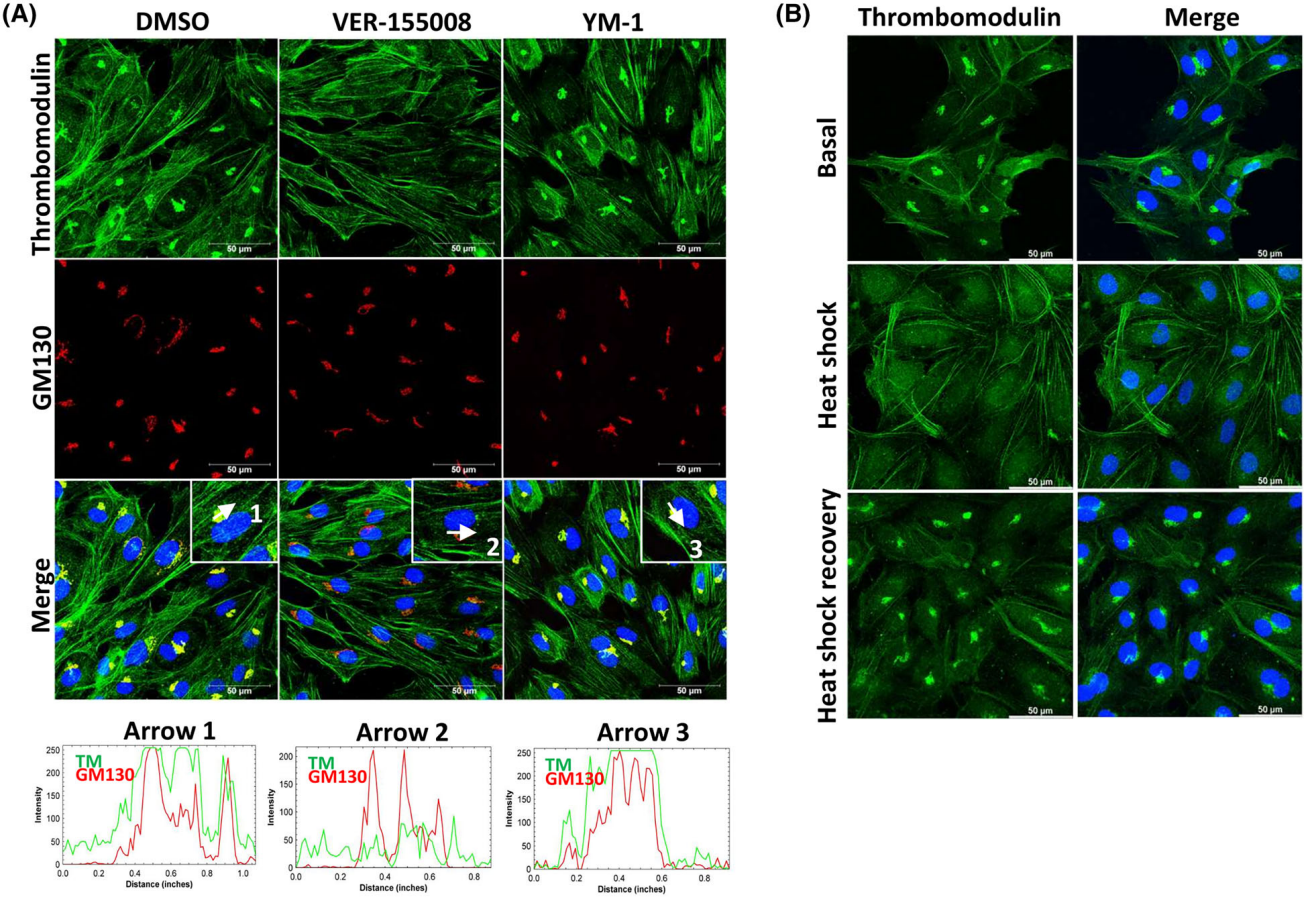
Thrombomodulin distribution modulated by HSP70 and heat shock response in endothelial cells. (A) Representative confocal immunofluorescence images of thrombomodulin TM (green; Invitrogen PA5‐21924), GM130 (red, BD Biosciences 610 822), nuclei (blue, Hoechst) of HUVEC after 24 h of 30 μm VER‐155008 and 20 μm YM‐1‐treated. Scale bars: 50 μm. Images are representative of three independent experiments using two different lot of cells (23TL213682 and 23TL086130). DMSO was used as a vehicle in basal cells. Histograms show plot profile intensities for TM and GM130 channels in the basal, VER‐155008, and YM‐1 treatments, indicated by arrows 1, 2, and 3, respectively. (B) Staining of TM (green, Invitrogen PA5‐21924) and nuclei (blue, Hoechst) after heat shock for 1‐h and 24‐h recovery. Representative images of *n* = 3 independent experiments using two different lots of cells (19TL028324 and 19TL023264). Scale bar = 50 μm.

Previously, we identified that endothelial thrombomodulin activity was negatively modulated by extracellular HSP70 [13]. However, the role of intracellular HSP70 was not investigated. Here, we show that the distribution of cell‐surface thrombomodulin was not affected (Fig. 4A). The ATP competitive inhibitor VER‐155008 blocked thrombomodulin trafficking to the Golgi complex, as evidenced by the loss of perinuclear thrombomodulin staining with GM130 compared to the vehicle control (Fig. 4A). A similar observation was made after endothelial heat shock (Fig. 4B), where HSP70 was upregulated [12]. During heat shock recovery, HSP70 is dedicated to binding and assisting unfolded/misfolded proteins to maintain proteostasis.

**Fig. 5:**
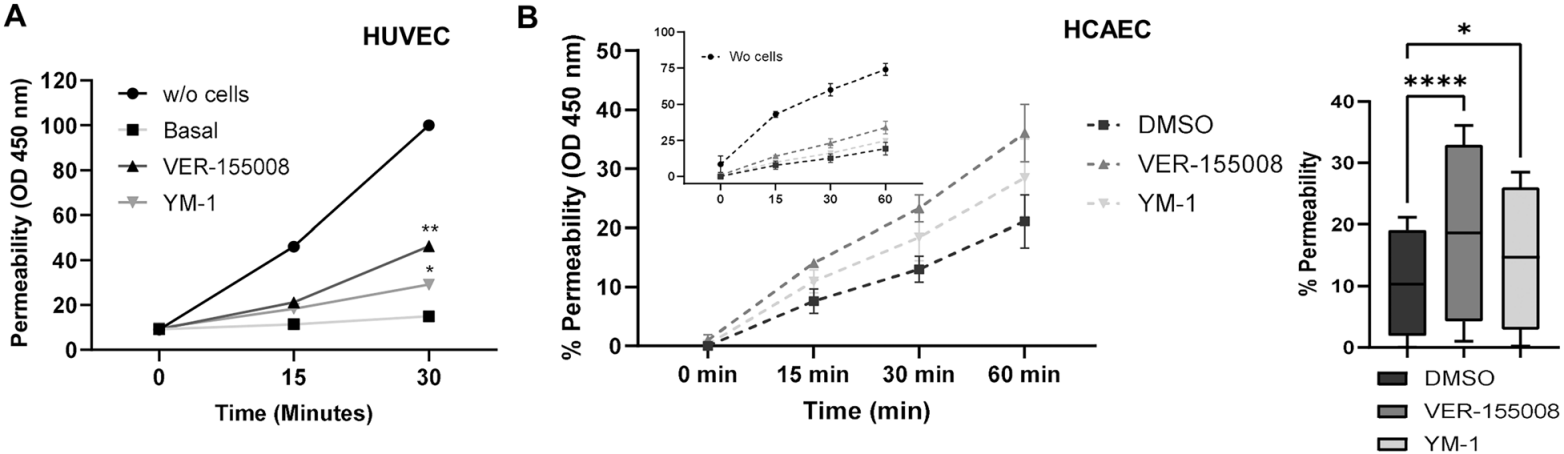
HSP70 loss of function impairs primary human vein and artery coronary endothelial paracellular flux. (A) HUVEC monolayer transwell‐seed was 50 μm VER‐155008, 40 μm YM‐1, or DMSO‐treated for 1 h in EBM‐2. The lower chamber IgG‐HRP (160 kDa) activity was measured, and data are presented as mean of three independent experiments (23TL213682 and 23TL135742), two‐way ANOVA, with Tukey's post hoc, *P < 0.05, **P < 0.01 versus Basal. (B) Permeability as a function of time of HCAEC (left) treated with DMSO (basal), 50 μm VER‐155008 or 40 μm YM‐1. An additional control without cells (100% permeability) is shown in the inset. Each point represents the mean ± SD of three independent experiments and an analysis of a two‐way ANOVA, with a Tukey's post hoc test, *P < 0.05, ****P < 0.0001 for statistical differences (right).

Another primary function of the endothelial monolayer, besides mechanotransduction, is to support tissue with metabolic demands and maintain appropriate vessel permeability [3]. VE‐cadherin, through homotypic interactions, contributes together with PECAM‐1 to the maintenance of vascular quiescence and integrity [2] and, in addition, is a component of the EC mechanosensory complex: PECAM‐1/CD31, VE‐cadherin, and VEGFR2 [7]. Then, we seek if HSP70, whose inhibition led to gaps between endothelial cells (Fig. 1D,E), could affect endothelium permeability. We discovered that vascular bed‐independent hyperpermeability occurs after pharmacological cytosolic HSP70 inhibition (Fig. 5A,B).

## Discussion

70 kDa‐ Heat shock protein is a central ATP‐dependent molecular chaperone that regulates vascular cell physiology, including migration, proliferation, and angiogenesis. However, specific clients have been underestimated. Here, we identified two new protein interactors of human HSP70 in endothelial cells: VE‐cadherin and PECAM‐1/CD31 (Figs 1, 2, 3). We demonstrate that HSP70 plays a central role in maintaining endothelial monolayer permeability and mediating the response to hemodynamic forces in primary human endothelial cells (Figs 1, 5). From a phenotypic perspective, cytosolic human HSP70 supports endothelial barrier integrity, mechanotransduction and regulates DNA synthesis (Fig. 1F,G). Thrombomodulin traffic to Golgi is affected by VER‐155008 (Fig. 4). Collectively, these findings suggest that HSP70 is a critical regulator of endothelial homeostasis, preventing dysfunction, as evidenced in cells isolated from patients with type IV hypertension [14] and consistent with observations in cardiovascular disease [1, 15].

Previous studies, including our own, have indicated that HSP70 plays a role in migration and angiogenesis [12, 14, 16, 18, 19, 32]; however, aside from tumor‐related angiogenesis [17, 32], specific endogenous intracellular HSP70 client proteins have remained largely undefined in vascular cells. Based on our findings, we suggest that intercellular transmembrane junction proteins, endothelial nitric oxide synthase (eNOS), and thrombomodulin (Fig. 1) may serve as substrates for HSP70. These observations may help to explain, at least from a mechanistic perspective, some of the vasculoprotective effects previously attributed to HSP70 [1, 14, 15, 33].

HSP70 interacts with thrombomodulin (TM) in endothelial cells [13], and inhibition of HSP70 ATPase activity impairs TM trafficking from the ER to the Golgi complex (Fig. 4A), supporting an essential role for HSP70 in thrombosis [33, 34], particularly under chronic conditions of intracellular HSP70 downregulation. Interestingly, HSP70 binds to and assists misfolded proteins, and TM trafficking during heat shock was similarly abrogated (Fig. 4B). Notably, intracellular and extracellular pools of HSP70 may exert opposing cardiovascular effects [13, 35]; intracellular HSP70, for example, protects cardiomyocytes under ischemic conditions mimicked by thermal stress [36]. Overexpression of HSF1 in human aortic endothelial cells has been shown to upregulate HSP70, eNOS, and thrombomodulin, while reducing plasminogen activator inhibitor‐1 (PAI‐1) expression and endothelin‐1 secretion [37]. We also observed that heat exposure increases HSP70 and HSP40 levels [12]. Notably, chronic heat shock in HUVEC has been reported to increase both thrombomodulin expression and activity [38], whereas severe heat shock may trigger elongation translation pausing through HSP70–ribosome interactions [39].

Our result, based on permeability, suggests an overlapping response in arterial and venous endothelial networks, whereas mechanotransduction phenotypes were specifically found in HUVEC. The first result coincides with another similar pathway found in the literature [40]. From a phenotypic perspective, several vascular diseases such as restenosis, atherosclerosis, hypertension, thrombosis, stroke, and diabetes are associated with impaired angiogenesis [41]. Understanding the difference between arterial and venous responses remains essential. We found that HSP70 inhibition disrupts cell‐surface distribution of mechanosensory components VE‐cadherin and PECAM‐1 in both venous and arterial endothelial cells (Fig. 1 D,E), correlated with altered permeability (Fig. 5A,B). We propose that HSP70 affects mechanosignaling by assisting PECAM‐1 folding and regulating VE‐cadherin turnover, likely through post‐translational modification‐dependent quality control. VER‐155008 impairs HSP70's role in translation by reducing heavy polysomes abundance in mammalian cells [39]. We also analyzed the primary structure of PECAM‐1 and VE‐Cadherin and found several potential HSP70‐binding motifs (Fig. 2E) based on consensus sequences recognized by DnaK [42]. Our data suggest that these junctional proteins may be client proteins of HSP70; however, additional studies are needed to confirm this, including the identification of specific binding sites and elucidation of the mechanistic regulation of PECAM‐1 and VE‐cadherin by the cytosolic HSP70 family.

Furthermore, we suggest that HSP70 modulates VE‐cadherin and PECAM‐1 intercellular junctions, contributing to an endothelial dysfunction characterized by impaired migration, angiogenesis, and vessel barrier function. PECAM‐1 is a crucial mechanosensor in the shear stress response [6, 7], highlighting HSP70 as a molecular hub in the biochemical signaling during mechanotransduction at least in vein (Fig. 1B). The disturbed alignment in the coronary artery was barely observed (Fig. 1C), which indicates that in arteries, other mechanosensory pathways continue to be active even in the condition of blocked HSP70. Although HSP70 is essential for maintaining vessel permeability, at the organismal level, the constitutive isoform HSC70 may compensate in its absence, as HSP70 knockout mice are viable [43]. Despite their high sequence identity/similarity (86%/93%), HSC70 and the inducible isoform (HSP70) display distinct enzyme kinetics [44] and engage different protein substrates in tumor cells [45].

A hallmark of endothelial cells is nitric oxide‐mediated vasorelaxation, which depends on endothelial nitric oxide synthase (eNOS) [46], and we observed that eNOS expression is decreased following HSP70 inhibition (Fig. 3). While our pharmacological approach does not discriminate between cytosolic HSP70 family members, future studies will address this challenge, especially considering the complexity of genetic manipulating primary endothelial cells. Inducible HSP70 knockout mice exhibit susceptibility to thrombosis in multiple models [33]; we propose that this may be partly explained by compromised endothelial barrier integrity resulting from the disruption of VE‐cadherin and PECAM‐1. We cannot rule out the possibility that HSP70 mediates post‐translational modifications in these junctional proteins.

Notably, VER‐155008 also binds endoplasmic reticulum HSP70 family member GRP78 [26]. However, given that the concentration of human cytosolic HSP70 is approximately three times higher than that of GRP78 [21], and considering that our assays were performed in the presence of serum‐containing medium (EGM2), it is unlikely that a substantial fraction of VER‐155008 would reach and inhibit GRP78. YM‐1 is an MKT‐077 analog considered selective for cytosolic HSP70 [27, 28]. Although it has been shown that MKT‐077 also binds mitochondrial HSP70 (encoded by the HSPA9 gene), YM‐1 has been predominantly localized to the cytosolic fraction in cancer cells [47], and its allosteric activity on cytosolic HSP70 is well characterized [28]. Despite this indirect evidence supporting target specificity, we cannot entirely rule out the possible contribution of another HSP70 family member to the observed effects. Overall, our findings in human vein and coronary artery primary endothelial cells support a central role for HSP70 in human vascular biology, and contribute to our understanding of how molecular chaperones, particularly HSP70 family, regulate phenotypic plasticity, which could be potentially relevant to the pathogenesis of cardiovascular and pulmonary diseases.

## Conflict of interest

The authors declare no conflict of interest.

## Author contributions

APM, EGM, ICBP, PVSO, and LBCT performed methodology, formal analysis, data curation, and validation. TLSA performed conceptualization, methodology, formal analysis, data curation, visualization, supervision, project administration, funding acquisition, and writing – original draft.

## Data Availability

All data supporting the findings of this study are included in this manuscript. The corresponding author, upon request, could share the noncropped western blotting.
